# Within-Host Evolution of the Dutch High-Prevalent *Pseudomonas aeruginosa* Clone ST406 during Chronic Colonization of a Patient with Cystic Fibrosis

**DOI:** 10.1371/journal.pone.0158106

**Published:** 2016-06-23

**Authors:** Rosa van Mansfeld, Mark de Been, Fernanda Paganelli, Lei Yang, Marc Bonten, Rob Willems

**Affiliations:** 1 Department of medical microbiology, University Medical Centre Utrecht, Utrecht, The Netherlands; 2 Novo Nordisk Foundation center for Biosustainability, Technical University of Denmark, Copenhagen, Denmark; Lee Kong Chian School of Medicine, SINGAPORE

## Abstract

This study investigates adaptation of ST406, a prevalent *P*. *aeruginosa* clone, present in 15% of chronically infected cystic fibrosis (CF) patients in the Netherlands, in a newly infected CF patient during three years using whole genome sequencing (WGS), transcriptomics, and phenotypic assays, including biofilm formation. WGS-based phylogeny demonstrates that ST406 is genetically distinct from other reported CF related strains or epidemic clones. Comparative genomic analysis of the early (S1) and late (S2) isolate yielded 42 single nucleotide polymorphisms (SNPs) and 10 indels and a single 7 kb genomic fragment only found in S2. Most SNPs and differentially expressed genes encoded proteins involved in metabolism, secretion and signal transduction or transcription. SNPs were identified in regulator genes *mexT* and *exsA* and coincided with differential gene expression of *mexE* and *mexF*, encoding the MexE/F efflux pump, genes encoding the type six secretion system (T6SS) and type three secretion system (T3SS), which have also been previously implicated in adaptation of other *P*. *aeruginosa* strains during chronic infection of CF lungs. The observation that genetically different strains from different patients have accumulated similar genetic adaptations supports the concept of adaptive parallel evolution of *P*. *aeruginosa* in chronically infected CF patients. Phenotypically, there was loss of biofilm maturation coinciding with a significant lower level of transcription of both *bfmR* and *bfmS* during chronic colonization. These data suggest that the high-prevalent Dutch CF clone ST406 displays adaptation to the CF lung niche, which involves a limited number of mutations affecting regulators controlling biofilm formation and secretion and genes involved in metabolism. These genes could provide good targets for anti-pseudomonal therapy.

## Introduction

*Pseudomonas aeruginosa* is a versatile Gram-negative rod with a relatively large genome of more than 6 million base pairs that can thrive in many different niches[1]. *P*. *aeruginosa* chronically infects the lungs of patients with cystic fibrosis (CF) and lung deterioration due to inflammation is the major cause of death in these patients[2]. More than half of the CF patients become chronically infected during their lifetime and most chronically infected patients harbor the same *P*. *aeruginosa* strain for many years or even life-long[3–5]. Several studies have investigated adaptation of *P*. *aeruginosa* trying to understand what the essential factors are for colonization and persistence in the lungs of CF patients despite the host immune system and aggressive antibiotic treatment[2;6–13]. Genome analysis of *P*. *aeruginosa* demonstrated that genes involved in biofilm formation, motility, transmembrane transport, hemolysis, secretion systems and resistance to oxidative stress and antibiotics are associated with CF lung adaptation [6]. Adaptation is mediated through remodeling of regulatory networks involving central metabolism and pathogenicity[13]. Phenotypic adaptation often involves a reduced growth rate, loss of motility, loss of substantial catabolic activities and inactivation of important regulatory functions[14]. Several studies demonstrated that parallel evolutionary events in these adapted strains do not seem to be due to loss or gain of certain genes[15], but rather to patho-adaptive mutations[10;13;16]. However, some CF adapted strains share accessory genomic elements like the LES-prophage-1, LESGI-2, and LESGI-4, that may contribute to increased competitiveness in the CF lung[17]. Adapted strains can be transmitted between CF patients and some of these epidemic *P*. *aeruginosa* clones seem associated with worse clinical outcome[18–23]. In the Netherlands, an epidemic *P*. *aeruginosa* clone with MLST type ST406, MLVA type CC27 and AT-chip single nucleotide polymorphism (SNP)-type A418 or E418 was found in up to 50% of CF patients between 15 and 25 years of age in 2007[24–26]. This clone was not found in clinical cultures from non-CF patients and was genotypically different from the epidemic CF strains found in other countries[26;27]. SNP-typing of clinical and environmental *P*. *aeruginosa* strains from all over the world only detected this genotype in Dutch CF patients[28]. The fact that this clone is specifically linked to the CF lung niche suggests that this genotype is very well adapted to the CF lung. In contrast to some other epidemic clones an association with clinical deterioration could not be detected for ST406[29]. Another clone (ST497) was found in 5% of Dutch CF patients and was associated with older age (>25 years)[25]. Likewise this genotype has thus far only been isolated from Dutch CF patients.

Insights into the evolutionary dynamics during chronic colonization of the lungs of CF patients and genetic relatedness of epidemic CF clones that are specifically adapted to the cystic fibrosis lung niche might give further clues for new eradication therapies or preventive measures. The aims of this study were to infer the phylogenomic relatedness of two high-prevalent *P*. *aeruginosa* clones in the Netherlands, ST406 and ST497, to a collection of *P*. *aeruginosa* strains and to describe genotypic and phenotypic changes of ST406 during adaptation to the CF airway and compare this to other known CF clones. To investigate the traits that may have contributed to adaptation of the *P*. *aeruginosa* ST406 clone to the CF lung we indexed genome-wide alterations by performing comparative genomics in combination with comparative transcriptomics and cataloging phenotypic differences between two ST406 strains obtained from the same patient, but separated by a timespan of three years. The first isolate was cultured one month after the patient became first colonized with *P*. *aeruginosa* (“early isolate” or S1) while the second isolate was recovered from the same patient after three years of chronic colonization (“late isolate” or S2).

## Methods

### Strains

The first, early, *P*. *aeruginosa* ST406 isolate (S1) was cultured from sputum from a 15 years old, Dutch CF patient in 2004, one month after the very first detection of *P*. *aeruginosa* in this patient. This patient became chronically infected after the first positive culture in 2004. The second, late, *P*. *aeruginosa* ST406 isolate (S2) was cultured in 2007 from sputum from the same CF patient. Both isolates were non-mucoid phenotype. ST497 isolate (S3) was cultured from another 26 year old, Dutch CF patient that had already been chronically infected with *P*. *aeruginosa* for more than seven years. Both patients were regularly visiting the University Medical Centre in Utrecht for their regular medical care. *P*. *aeruginosa* strain PA01 and *E*. *coli* strains that harbor *lasB-gfp* (MH155) or *rhlA-gfp* fusion for quorum sensing detection were kindly provided by Søren Molin from DTU, Denmark.

### Whole genome sequencing and SNP analysis

Genomic DNA was extracted from *P*. *aeruginosa* isolates S1, S2 and S3 using a Wizard Genomic DNA Purification kit (Promega). Whole genome sequencing was performed on an Illumina MiSeq platform. Raw 2×250 bp paired-end Illumina reads were quality-filtered using Nesoni 0.109 [https://github.com/Victorian-Bioinformatics-Consortium/nesoni] (with following options: adaptor-clip (yes), adaptor-match (10), adaptor-match max error (1), clip ambiguous (yes), quality cutoff (10), minimum read length (150)). De novo assembly was done using SPAdes 2.5.1[30]: kmers used (21, 33, 55, 77, 99, 127), with “careful” option turned on and cutoffs for final assemblies: minimum contig/scaffold size = 500 bp, minimum contig/scaffold average Nt coverage = 10-fold. Gene prediction and annotation was done using Prokka[31] (default options; using a Pseudomonas-specific BLAST database, as provided in the Prokka package) and RAST[32]. Additional functional annotation of protein-coding genes was done using the COG database[33]. Protein sequences were queried against the COG myva database using BLAST 2.2.29+[34] and received the same COG as their best BLAST hit (E-cutoff ≤ 1e-10). Sequencing reads have been submitted to the European Nucleotide Archive under study PRJEB12885.

To compare differences between the ST406 S1 and S2 isolates and investigate within-patient adaptation an in-house read-mapping pipeline was used to detect SNPs and small indels. Nesoni-filtered reads of S2 were mapped against the S1 assembly using Bowtie2[35]. To filter for genomic repeats, we removed reads that mapped to multiple positions in the S1 assembly. SAMtools 0.1.18[36] was used to call SNPs and indels with following conditions: Qscore ≥ 50, mapping quality ≥ 30, and calls were required to be homozygous (under diploid model). Additional criteria included a mapping depth ≥ 10 reads, a consensus of ≥ 75% to support a call, and ≥ 1 supporting read in each direction. To correct for potential assembly errors, we also performed the SNP/indel-calling procedure described above after mapping Nesoni-filtered reads of S1 against its own assembly. Genomic positions containing SNPs and indels in the S1 vs S1 comparison were ignored in the S2 vs S1 comparison. Identified SNPs and indels were linked to features (i.e. genes) and were inspected for synonymous vs non-synonymous mutations (in case of SNPs).

To compare gene content between S1 and S2 we used Inparanoid v4.129. All S1 genes that did not have a predicted orthologous relationship with an S2 gene and vice versa were considered as potentially unique for that given strain. To further verify these potential strain-specific genes, S2 reads were mapped against the S1 assembly and vice versa as described above, but without filtering for reads mapping to multiple genomic positions. Using the same criteria described above for finding SNPs and small indels, genomic regions significantly covered by mapped reads were identified. Potentially strain-specific genes identified using Inparanoid were considered to be truly strain-specific if ≤ 10% of their entire length was covered by reads.

### Core genome alignment

To investigate phylogenetic relatedness of the three *P*. *aeruginosa* isolates from Dutch CF patients that represent two prevalent Dutch clones with an international collection of *P*. *aeruginosa* strains all publicly available *P*. *aeruginosa* whole genome sequences (WGSs) were downloaded from GenBank on 12 February 2014. The genome sequences of these publicly available strains and of strains S1, S2 and S3 were aligned using an in-house pipeline that makes use of the NUCmer v3.23 alignment algorithm[37]. All genomes were aligned against the completed genome sequence of *P*. *aeruginosa* PA01 (i.e. used as reference strain). Repetitively aligning genomic regions were removed from each pairwise alignment after which all pairwise alignments were merged into one *P*. *aeruginosa* core genome alignment. A phylogenetic tree was built from the polymorphic sites in the core genome alignment using RAxML[38]. The tree was built under the GTR model. Confidence was inferred by running 1000 bootstrap replicates under the same model. The tree was visualized using MEGA6[39]. Odds ratio’s for calculating significance of associations between source (CF sputum isolate, clinical isolate, or environmental isolate) and clade assignment was done using MedCalc software (https://www.medcalc.org/calc/odds_ratio.php). The *P*-value threshold for statistical significance was set at 0.05.

### Transcriptome analysis

Transcriptome analysis using GeneChip *P*. *aeruginosa* genome arrays (Affymetrix) was performed as described previously[14]. *P*. *aeruginosa* strains were grown aerobically in Luria-Bertani (LB) medium starting from OD600 = 0.01 and harvested at OD600 = 0.5. RNA was isolated using RNeasy Mini Purification kit (QIAGEN) and transcribed into cDNA using random primers (Invitrogen Life Technologies). Subsequently, cDNA was purified (QIAquick, QIAGEN), fragmented and labeled and hybridized on an Affymetrix *P*. *aeruginosa* PA01 gene chip. The probe arrays were scanned with a GeneChip Scanner 3000 and the raw data was obtained using the Affymetrix GeneChip Operating System 1.4. Microarray data analysis was performed as described before[24] using bioconductor in R environment (http://www.bioconductor.org). Normalization and expression index calculation was done with rma function[40]. The fold change was calculated using the average expression levels of three replicates. A cut off p-value < 0.05 and fold change > 2 between transcriptome levels of S1 and S2 was considered significant. The annotations and functional classes were assigned according to the Pseudomonas Genome Database[41].

### Amplification of S2 specific genes

To verify differences in gene content between S1 and S2 (detected by comparing whole genome sequence data as described above) and analyze presence of these genes in other *Pseudomonas aeruginosa* isolates we performed PCR amplification in 29 non-ST406 clinical isolates (6 ICU, 6 community acquired, 6 CF, 6 other clinical isolates from a previous study[26] and 3 isolates of the Liverpool epidemic PA01 and the Midlands epidemic strain) (S1 Table). Isolates were cultured on tryptic soy agar (TSA)-blood plates (Becton, The Netherlands) overnight at 37°C, suspended in 20 μl lysis buffer (0.25% (wt/vol) SDS, 0.05 N NaOH) and incubated at 95°C for 20 min. The cell lysate was centrifuged and diluted with 180 μl buffer (10 mM Tris-HCl, pH 8.5). After thoroughly mixing, another centrifugation for 5 min at 16,000 x g was performed to remove cell debris. Supernatants were frozen at -20°C until further use. Two μl of the lysate was used in a touchdown PCR using Hotstar Taq DNA polymerase (Qiagen Benelux B.V.), and 5 μl Q-buffer (Qiagen Benelux B.V., Venlo, the Netherlands). The PCR was conducted as follows: 10 min at 96°C, then 10 cycles of 30 s at 95°C, 30 s at 65°C with 1°C less every cycle and 1 min at 72°C. This was followed by 25 cycles of 30 s at 95°C, 30 s at 55°C and 1 min at 72°C, after which the final step of 10 min at 72°C followed. Presence of PCR products was checked by electrophoresis on 1% (wt/vol) agarose gel. S2 was used as positive control. Primers used for PCR amplification of S2 specific genes and non-ST406 *P*. *aeruginosa* strains used to test presence of these genes are shown in S1 Table.

### Phenotypic experiments

Phenotypic traits known to be important virulence factors that are involved in adaptation to chronic infection of the CF lung are growth rate, excreted proteases, motility, quorum sensing and biofilm formation were compared between S1 and S2. PA01 was used as control.

#### Motility assays

Swimming, swarming and twitching motility and protease assays were performed in triplicate. Bacteria were grown overnight on LB agar plates. Swimming, swarming and twitching motility assays were performed on respectively 0.3% (wt/vol), 0.6% (wt/vol) and 1.5% (wt/vol) agarose plates containing Agrobacterium (AB) minimal medium with glucose (0.5%(wt/vol)) and casamino acids (0.5% (wt/vol)). One bacterial colony was inoculated in, on and through the agar by sterile toothpick, respectively. Swimming ability was assessed after 24 h incubation at 30°C by measuring the maximum diameter of the zone of growth. Swarming and twitching was assessed after 48 h of incubation at 37°C by measuring zones of growth. Protease production in skim milk was measured by inoculating 100 μl of supernatant of overnight culture (after spinning at 7000 g for 5 min) on a LB-agar plate with 10% (wt/vol) skim milk concentration. Clear zones of protease production were measured after 48 h of incubation at 37°C.

#### Growth curves

Growth curves were made by measuring OD600 every 50 min, starting with an OD600 of 0.01. Doubling time was calculated in Microsoft Excel using the exponential trend line (tD = (ln2/μ)).

#### Quorum sensing

Quorum sensing signal production was tested by streaking isolates and a positive and negative control near two *E*. *coli* monitor strains that harbor *lasB-gfp* (MH155) or *rhlA-gfp* fusion products on a plasmid[42] that can be induced by the Las (3-O-C12-HSL) and Rhl signal molecule (C4-HSL), respectively. GFP (green fluorescent protein) production was visualized after 24 hincubation at 37°C using a Zeiss Axion2 microscope with an integrated Coolsnap color cf camera at 2.5x magnification and 200ms exposure time.

#### Phenotype MicroArrays

To investigate difference between S1 and S2 in growth under conditions using different nutritional sources and other chemical compounds like toxins, phenotypic microarrays were performed. Quantitative measurements of 1821 phenotypes of S1, S2 and of *P*. *aeruginosa* strain PA01 were determined using phenotype MicroArrays (Biolog, Inc Hayward CA, USA). In this assay cell respiration, measured by color change caused by reducing a tetrazolium dye, is used as a universal reporter for both amplification and precise quantitation of phenotypes. The measured average height of two replicates of each phenotype was compared between S1 and S2. Phenotypes with more than two-fold difference were considered different phenotypes. Among the 1821 phenotypes tested by this method were 190 Carbon-sources, 384 Nitrogen-sources, 60 Phosphor- sources, 192 phenotypes related to osmolytes and pH and 960 to chemical sensibility.

#### Antibiotic susceptibility and colony morphology

Susceptibilities were determined by disk diffusion on Muller Hinton plates using EUCAST breakpoints for ciprofloxacin, levofloxacin, piperacillin, ceftazidime, tobramycin, amikacin, meropenem, piperacillin/tazobactam, colistin trimethoprim, trimethoprim/sulfamethoxazole, chloramphenicol.

Colony morphology was checked under the plate microscope.

#### Biofilm experiments

A semi-static biofilm model was used to assess biofilm formation of S1 and S2, as described before[43]. Overnight bacterial cultures were diluted to an OD660 of 0.01 in 6 ml LB medium with 1%(wt/vol) glucose and added to a coverslip coated with poly-L-lysine (0.45 μm; diameter, 12 mm; Becton Dickinson) inside a well from a six-well polystyrene plate (Corning Inc.). Biofilms were grown at 30°C for 48 hat 120 rpm. After 48 h, the coverslips were washed with 0.85% (wt/vol) NaCl and the biofilms were chemically fixed with 8% (vol/vol) glutaraldehyde (Merck) for 20 min. Subsequently, the biofilms were stained with 15 μg/ml propidium iodide (PI) in 0.85% (wt/vol) NaCl that was removed after 15 min. The coverslips were transferred to glass microscope slides and analyzed by a confocal laser scanning microscope (CLSM) (Leica SP5), equipped with an oil plan-Neofluar ×63/1.4 objective. PI was excited at 488 nm. Z stacks were taken with an interval of 0.42 μm. Pictures were analyzed with LAS AF software (Leica), and biofilm thickness and biomass were quantified using Comstat[44]/Matlab R2013b software (the MathWorks). The average thickness and biomass of the biofilms were measured at ten randomly chosen positions and statistical significance determined by unpaired two-tailed Student's t-test. Experiments were performed twice in duplicate.

## Results and Discussion

### Whole genome sequencing and phylogenetic inferences

**Fig 1:**
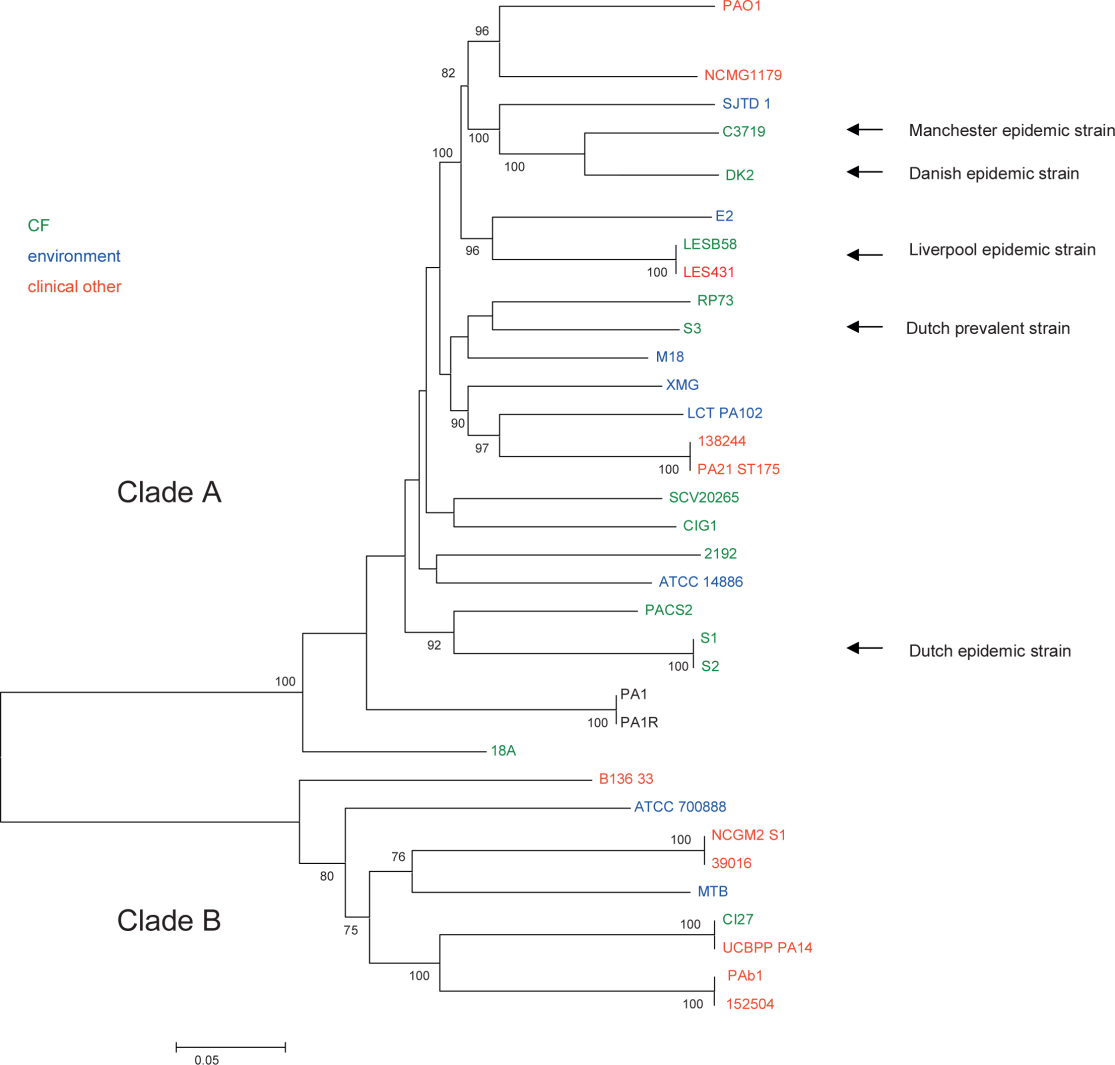
Phylogenetic tree including 34 *P*. *aeruginosa* strains built from an alignment of 121k core SNPs. Numbers along the branches indicate bootstrap values and only bootstrap values ≥75% are shown. Branch lengths correspond to the scale bar, in units of changes/nucleotide position. Green = CF sputum isolate, red = clinical isolate, not CF, blue = environmental isolate. The Dutch high-prevalent CF strains and internationally known epidemic CF strains have been indicated.

To determine the phylogenetic relationship between Dutch prevalent clones, ST406 (S1 and S2) and ST497 (S3), and other epidemic clones whole genome sequencing was performed. Illumina sequencing and subsequent assembly of the three *P*. *aeruginosa* isolates S1, S2 and S3 yielded draft genomes with an average assembly size of 6.32, 6.32, and 6.28 Mbp, consisting of 45, 42, and 48 scaffolds with a nucleotide coverage of 56.4, 55.9 and 81.1, respectively. To assess the phylogenetic relatedness of the two Dutch epidemic clones, represented by the three *P*. *aeruginosa* isolates from Dutch CF patients S1, S2 and S3, with an international collection of *P*. *aeruginosa* strains, publicly available genome sequences of 37 *P*. *aeruginosa* strains were downloaded and aligned together (S2 Table). This resulted in a core genome alignment of 3.27 Mb, containing 258,000 SNPs. An initial SNP-based phylogenetic tree showed that strains PA7 & VRFPA01 clustered far away from the other *P*. *aeruginosa* strains. To increase resolution, PA7 & VRFPA01 were left out and the remaining 38 strains were re-aligned. Identical strains were removed from the tree for clarity (MPA01P1, MPA01P2 and PA0579 represented by PA01 and PADK2CF510 represented by DK2). The final core genome alignment was 4.02 Mb and a phylogenetic tree including 34 strains was constructed using the 121,244 SNPs contained in this alignment (Fig 1).

The phylogenetic tree demonstrated that the Dutch high-prevalent *P*. *aeruginosa* CF strains, S1/S2 and S3 were evolutionary not closely related to other epidemic CF related clones. Also the Liverpool epidemic strain clustered separately, while the Manchester and Danish epidemic CF strain shared a common ancestry. Two distinct clades (A and B) were identified in the tree, which contain both environmental and clinical isolates as well as isolates from CF patients. However, clinical non-CF isolates were significantly associated with clade B (OR: 8.0; 95% CI: 1.5–43.7; *p*<0.01), while CF isolates were enriched in clade A, although this was not significant (OR: 7.4; 95% CI: 0.8–68; *p* = 0.08). Even though, there seems to be a trend towards significance and a larger number of isolates would be required to investigate the finding further and these results could suggests that clinical non-CF isolates may have an evolutionary background that is distinct from isolates colonizing CF patients. Of course, numbers of strains in our study are relatively small, and selection of strains that were chosen for WGS might contain a bias.

In contrast to these observations, several studies investigating both clinical and environmental isolates have demonstrated that *P*. *aeruginosa* has a non-clonal epidemic population structure with no clear association between specific lineages and certain niches[23;28;45–50]. However, these studies indexed only a limited number of loci providing limited power for inferring phylogenetic relationships. The fact that not all epidemic CF isolates group in one lineage indicates that these CF isolates do not form one monophyletic group, thus do not share a recent common ancestor. Clearly, the Manchester, Danish, Liverpool, and both Dutch epidemic CF strains form a polyphyletic group in which phylogenetically different sequences have converted into a successful epidemic phenotype in CF patients.

### Comparative genomics and transcriptomics of the early (S1) and late (S2) ST406 isolate

**Fig 2:**
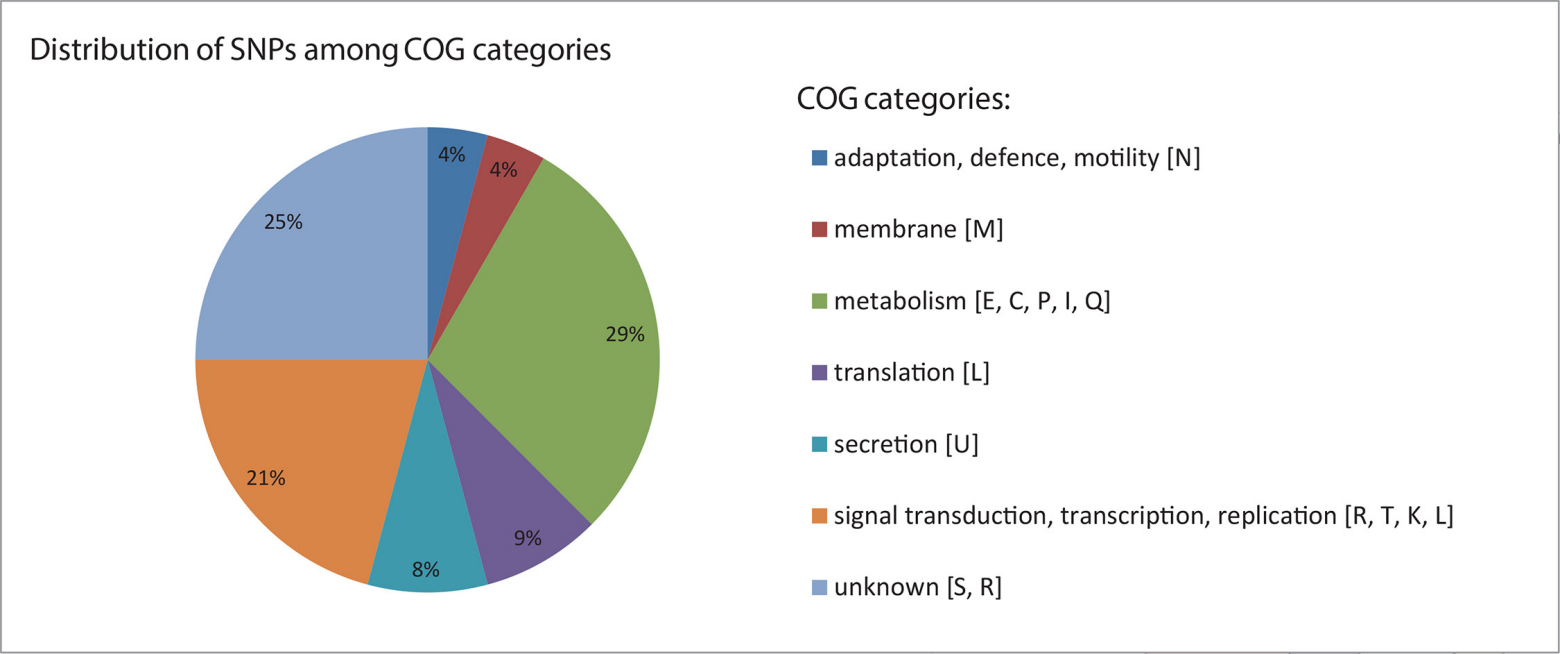
COG categories of non-synonymous SNP differences found between S1 (early isolate) and S2 (late isolate) isolated three years apart from the same patient. Percentage per category of total non-synonymous SNPs is indicated.

To investigate genomic differences between S1 and S2, 6,284,869 bases (99.4–99.5% of total assembly size) of the two isolates were aligned and this yielded 42 high quality SNPs and 10 indels. Twelve SNPs were synonymous, while 24 were non-synonymous (Table 1). Five SNPs were located in intergenic regions and one occurred in an rRNA encoding gene. Most (29%) non-synonymous mutations were in genes coding for COG categories involved in metabolism, followed by non-synonymous SNPs in COG category transcription and signal transduction (21%) and category unknown (25%) (Fig 2).

**Table 1:** 24 non-synonymous SNPs detected between early (S1)and late (S2) ST406 isolate.

ST 406 gene	SNP [aa change]^a^	amino acid position	PA01 annotation	gene	COG function classification	SNP in same gene in literature
3848	AAC[N] > ATC[I]	63	PA3545^b^	*algG*	Inorganic ion transport and metabolism	[9], [13], [14], [53]
5027	AAC[N] > ATC[I]	178	PA4102	*bfmS*	Signal transduction mechanisms	[13], [14]
3581	AAG[K] > GAG[E]	297	PA1782		General function prediction only, Signal transduction mechanisms, Transcription, Replication, recombination and repair	[13]
5598	AAT[N] > GAT[D]	41	PA4276	*secE*	Intracellular trafficking, secretion, and vesicular transport	[13]
5091	ACC[T] > GCC[A]	460	PA4039		Cell motility	[13]
1522	ACG[T] > GCG[A]	1063	PA2727 + PA2728		Replication, recombination and repair	[13]
4626	AGC[S] > AGA[R]	224	PA2385^b^	*pvdQ*	General function prediction only	[13]
4879	ATC[I] > AGC[S]	3973	PA0690		secretion	[13], [14]
0345	ATC[I] > GTC[V]	456	PA1147		Amino acid transport and metabolism	[13]
2241	ATG[M] > AAG[K]	86	PA4519	*speC*	Amino acid transport and metabolism	[9]
3651	CCG[P] > CTG[L]	156	PA1713	*exsA*	Transcription	[8],[9],[10], [13], [53]
5490	CTG[L] > ATG[M]	30	PA4163		Translation, ribosomal structure and biogenesis	[13]
0807	GCC[A] > ACC[T]	703	PA0794		Energy production and conversion	[13]
3973	GCC[A] > GGC[G]	61	PA3280	*oprO*	Inorganic ion transport and metabolism	[13]
5588	GCC[A] > GTC[V]	489	PA4266^b^	*fusA1*	Translation, ribosomal structure and biogenesis	[10], [13], [53]
2194	GCG[A] > GTG[V]	15	PA4562		General function prediction only	[13]
0321	GGC[G] > GTC[V]	118	PA1171		Cell wall/membrane/envelope biogenesis	[13]
4666	GTC[V] > ATC[I]	204	PA2346		Lipid transport and metabolism	[13]
4610	GTC[V] > GCC[A]	1523	PA2402		Secondary metabolites biosynthesis, transport and catabolism	[13]
4794	GTC[V] > GCC[A]	80	PA2552		Lipid transport and metabolism	[13]
2089	TAG[stop] >TGG[W]	51	PA4661	*pagL*	no COG	[13], [14]
4856	TTC[F] > TTG[L]	34	PA2492^b^	*mexT*	Transcription	[8], [13]
0194	TTG[L] > TTT[F]	68	PA1300		Transcription	[13]
5101	TTG[M] > CTG[L]	213	PA4029		Function unknown	[13]

^a^ A = Alanine, D = Aspartic acid, E = Glutamic acid, F = Phenylalanine, I = Isoleucine, K = Lysine, L = leucine, M = methionine, N = asparagine, P = proline, R = arginine, S = serine, T = treonine, V = valine, W = tryptophan. SNPs are indicated within their codon context, in the order: codon-S1 > codon-S2.

^b^ pathoadaptive gene by Marvig *et al* or Winstanley *et al*.[10;51]

Metabolic alterations and changes in regulatory functions have also been described for other CF adapted clones[13]. Whole genome sequencing of various isolates from different CF patients that harbored the DK2 clone for many years revealed loss of catabolic activities and inactivation of important regulatory systems[14]. Furthermore, analysis of non-synonymous SNPs in core genes of 32 CF related strains including LES (Liverpool epidemic strain) isolates revealed SNPs in genes encoding proteins involved in oxidoreductase activity, secretion and heterocycle metabolism[6].

**Fig 3:**
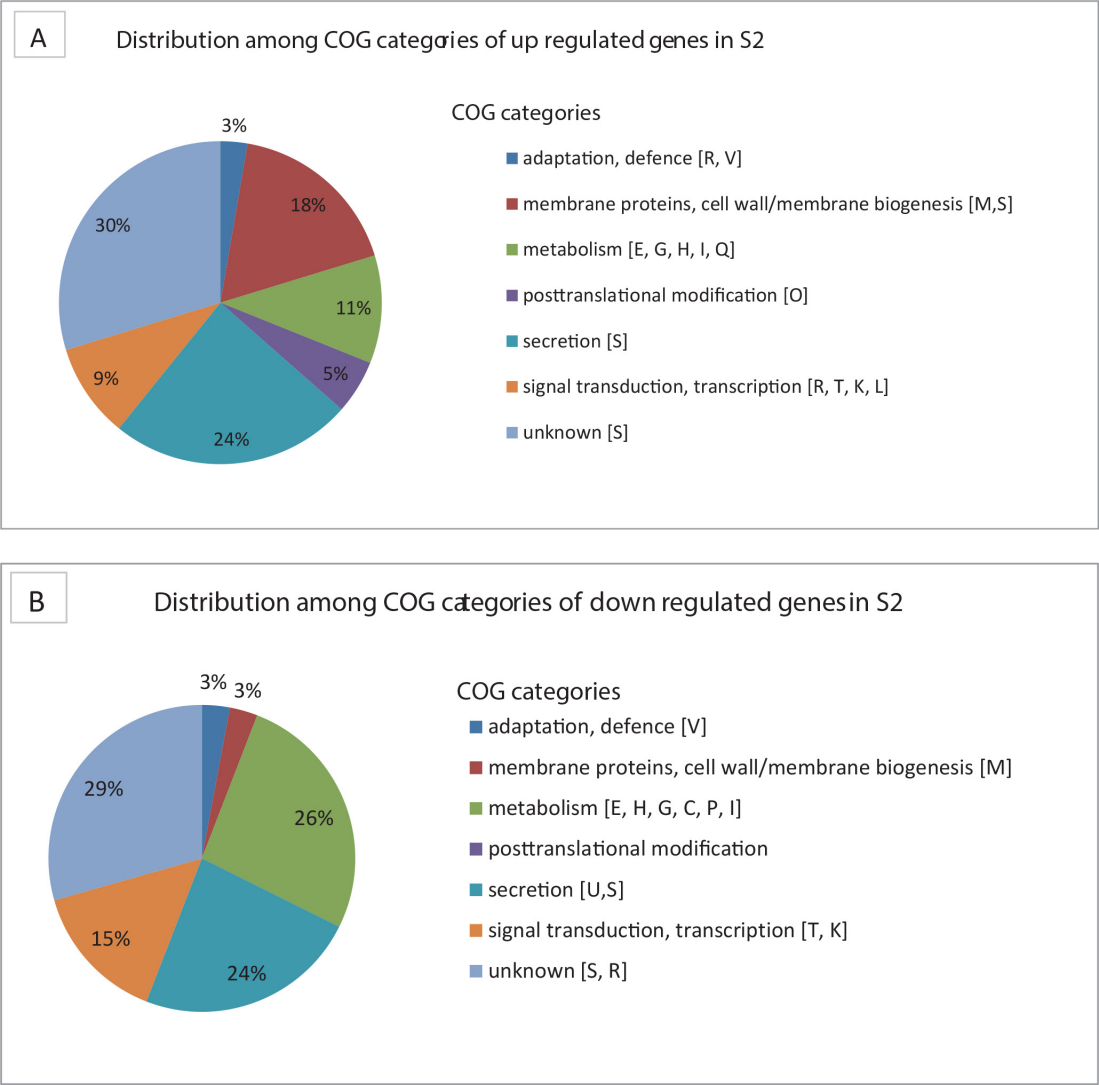
Functional categories of up- and down-regulated genes in S2 relative to S1 detected with Affymetrix transcriptome analysis. A, indicates the percentage of genes per COG category that were higher expressed in S2 compared to S1, while B, indicates the percentage of genes per COG category that were lower expressed in S2 compared to S1. COG category is indicated between brackets.

Comparative transcriptomics between ST406 S1 and S2 yielded 179 genes with different transcription levels (p< 0.05), of which 110 genes had a more than 2-fold difference in gene expression (S3 Table). Gene ontology enrichment analysis revealed overrepresentation of differentially expressed genes involved in metabolism and posttranslational modification that were higher expressed in S1 and genes encoding membrane proteins or involved in membrane or cell wall biogenesis and proteins involved in secretion that were higher expressed in S2 (Fig 3). Approximately 92% of all the genes in strains S1 & S2 were covered by uniquely and perfectly matching probes on the Affymetrix *P*. *aeruginosa* PA01 GeneChip when using a cutoff of at least 4 probes per gene (~96% in PAO1). This indicates that only a small proportion of S1 and S2 genes was not analyzed for transcription differences.

All SNPs were located in genes that have been previously described in adaptation of *P*. *aeruginosa* to the lung of CF patients and of which some were designated pathoadaptive genes (Table 1). These SNPs were in the same genes but not identical to other published SNPs. SNPs and transcriptome differences between S1 and S2 in genes that were deemed important for observed phenotypic differences are described in more detail below.

Recent studies have shown that in one sputum sample of a CF patient a variety of *P*. *aeruginosa* isolates of the same genotype with variable phenotypes and based on WGS data several SNPs can be detected[7;13;51–53]. This suggests that in these niches *P*. *aeruginosa* behaves as a quasispecies, *i*.*e*. a large group or cloud of related genotypes that exist in an environment of high mutation rate[54]. This adaptive radiation can explain why in various longitudinal studies in CF patients, in addition to common parallel evolutionary events in chronically colonizing *P*. *aeruginosa* isolates, also different unique sets of putative adaptive SNPs and phenotypes have been identified.

#### ExsA and the type III secretion system (T3SS)

A non-synonymous SNP (change from P156L in isolate S2) was identified in *exsA* (Table 1). Furthermore, one nucleotide insertion was detected upstream in the promoter region of this gene (Table 2). ExsA is a member of the AraC family of transcriptional regulators and is the primary regulator of the type III secretion system (T3SS). ExsA controls expression of genes implicated in T3SS biogenesis by directly binding to promoter sequences of these genes leading to activation of transcription[55]. In correspondence with these findings, our transcriptome analysis indicated differential expression of T3SS genes, with a significantly lower level of expression in isolate S (S3 Table). Also *exoS*, encoding the ExoS toxin, which is secreted by T3SS was expressed at a lower level in S2. Type III secretion mediated translocation of exoenzymes is used by *P*. *aeruginosa* to deliver exoenzyme effector molecules, like ExoS, into the eukaryotic cell. Our findings of higher expression of genes encoding T3SS in S1 relative to S2 suggest that T3SS may play an important role in acute CF infection but not or to a lesser extent during chronic infection. This is consistent with previous findings that also demonstrated down regulation of ExoS in chronically infected CF patients[56]. On the other hand, a recent report also found up-regulation of *exoS* during chronic infections[57]. These conflicting data indicate that further studies are needed to elucidate the exact role of ExoS during acute and chronic infections.

**Table 2:** Indels in isolate S2 relative to isolate S1.

Indel in S2	PA01 annotation	gene	Gene product annotation
GC > GCC (+1C)	PA1302		putative heme utilization protein
GGC > GCGGTCCTGCAACTGC (+13CGGTCCTGCAACT)	PA4967	*parE*	topoisomerase IV subunit B
GCTGCGGCGC > GCTGCGGCGCGGCCGCCTGCGGCGC (+15CTGCGGCGCGGCCGC)	PA2727		histidine kinase
TGGG > TGG (-1G)	PA4661	downstream of *pagL*	lipid A 3-O-deacylase
GGGT > G (-3GGT)	PA4379		methyltransferase domain-containing protein
TGGTAGGTA > TGGTA (-4GGTA)	PA3806		putative Fe-S-cluster redox enzyme
TGCCGGCCG > TGCCG (-4GCCG)	PA0347	downstream of *glpQ*	glycerophosphoryl diester phosphodiesterase
T > TG (+1G)	PA1713	upstream of *exsA*	AraC family of transcriptional regulators
GT > G (-1T)	PA2172		putative cellulase
CT > C (-1T)	PA0705	*migA*	alpha-1,6-rhamnosyltransferase

#### MexT, the MexEF efflux pump and the type VI secretion system (T6SS)

Another non-synonymous SNP (change from F34L in isolate S2) was located in *mexT*, which encodes the transcriptional regulator MexT that positively regulates the MexEF-OprN efflux pump and negatively regulates genes encoding T6SS[58–61] (Table 1). Transcriptome analysis revealed that the *mexEF* genes encoding the MexEF efflux pump were expressed at a higher level in the early S1 isolate, while genes encoding T6SS were expressed at higher levels in S (S3 Table). This suggests that the non-synonymous mutation in *mexT* affects some of the effector functions of MexT. The Mex E/F-oprN efflux operon also confers resistance to quinolones, chloramphenicol and trimethoprim. Down-regulation of *mexEF* genes in S2 coincided with less resistance against chloramphenicol and trimethoprim/ sulfa-methoxazole, while both isolates are resistant to quinolones and trimethoprim (S4 Table). It is interesting to note that *mexT* has been described as a mutational “hot spot,” where mutations can contribute to global phenotypic changes in *P*. *aeruginosa*[62].

#### MigA, PagL and LPS

**Fig 4:**
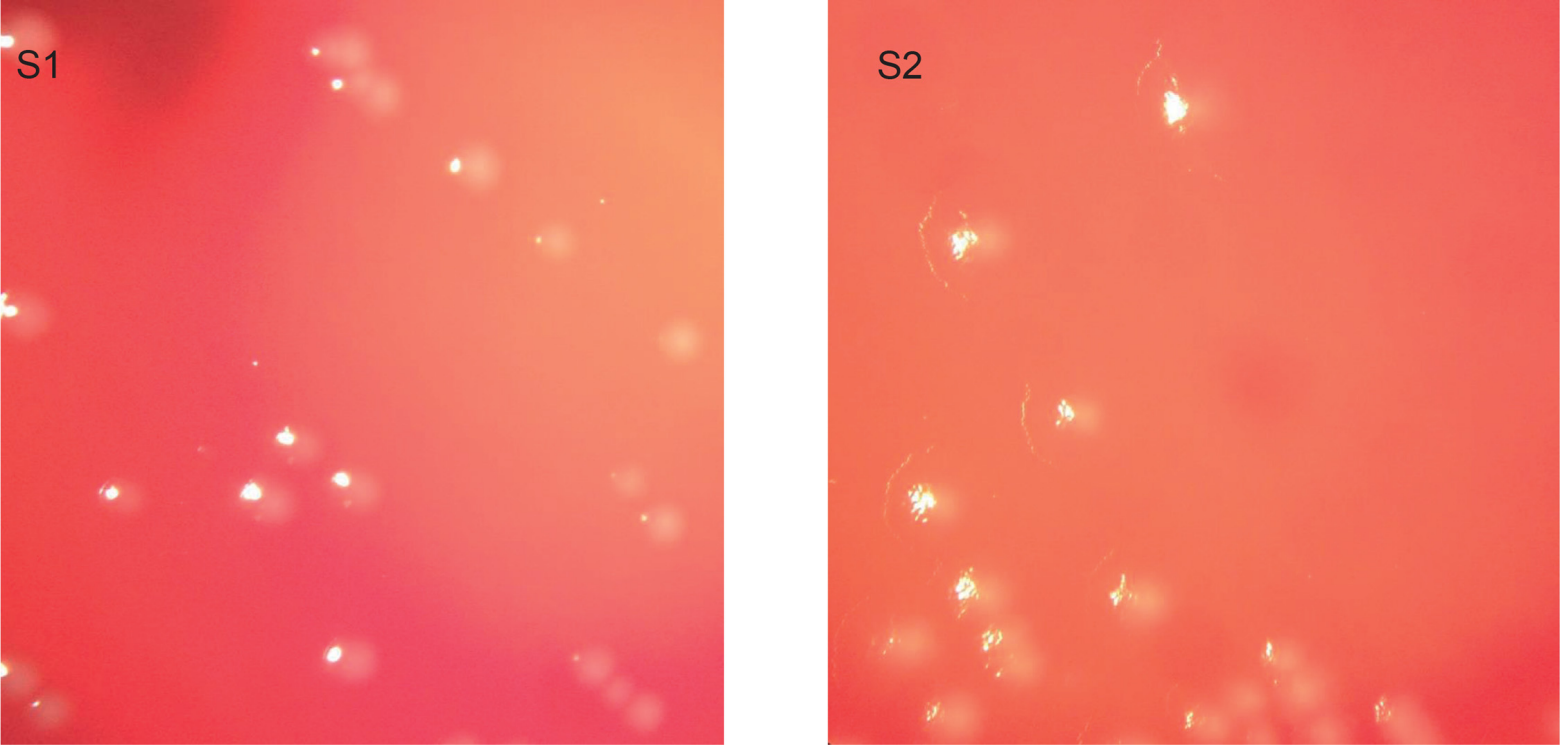
Colony morphologies on sheep blood agar. S1 (left) has a smooth colony morphology, while S2 (right) has a more rough colony morphology.

The early and late isolates displayed different colony morphology with a rougher colony morphology of S2 compared to S1 (Fig 4). Interestingly, a single nucleotide deletion in S2 relative to S1 and a non-synonymous SNP were detected in two genes, *migA* and *pagL* respectively, implicated in lipopolysaccharides (LPS) biogenesis. Furthermore, S2 contains a single nucleotide deletion just downstream of *pagL*, which may explain the observed differences in colony morphology (Tables 1 and 2). The *migA* gene encodes an Alpha-1,6-rhamnosyltransferase involved in producing uncapped core oligosaccharide[63] while *pagL* encodes a lipid A 3-O-deacylase, which recognizes either 3-OH C10 or 3-OH C14 moieties of the lipid A component of LPS and adjusts the structure of lipid A. Mutations in genes affecting LPS biogenesis have also previously been described in *P*. *aeruginosa* during CF lung adaptation[9;10;14]. In *P*. *aeruginosa* isolates from CF respiratory infections the O-antigen is not produced in high amounts. Since O-antigen is highly immunogenic this adaptation possibly facilitates chronic persistence[12]. PagL is overexpressed in *P*. *aeruginosa* isolates isolated from CF infants compared to those from acute infections or environmental isolates. However, it has also been shown before that in chronic infection with severe lung disease PagL function is lost[64]. Together with our findings this indicates that LPS is under selective pressure in the CF lung and that LPS modifications contribute to adaptation of *P*. *aeruginosa* to the CF airway.

### Biofilm formation and comparison of other phenotypic differences

**Fig 5:**
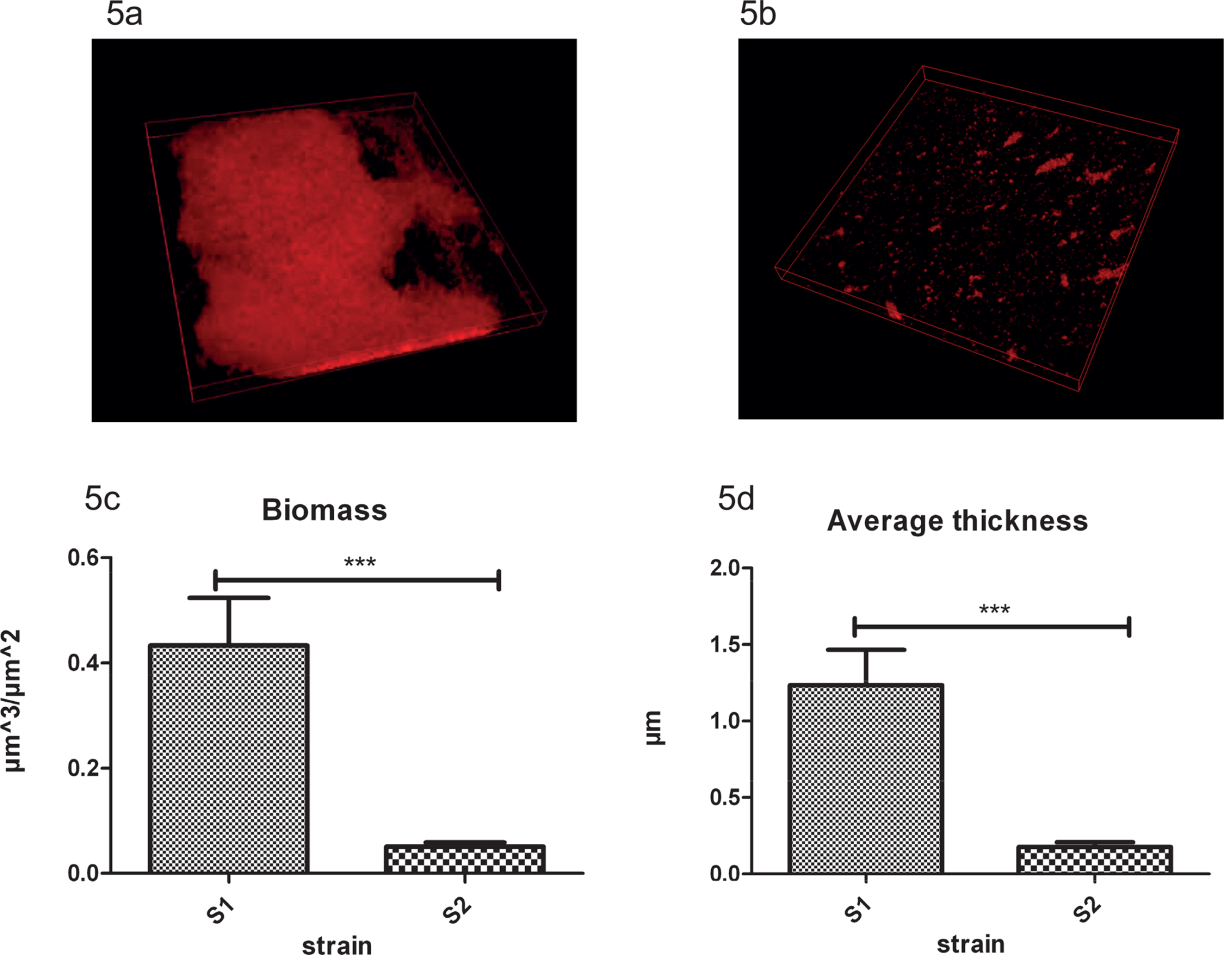
**Biofilm formation**. Biofilm formation in semi-static model of S1 (a) and S2 (b) after 48 h in LB+1% (wt/vol) glucose; Difference in total biomass (c) and average thickness (d) with Standard deviation error bars. Asterisks represent significant differences (*** P<0.005) as determined by an unpaired two-tailed Student's t-test.

Analyzing biofilm formation of S1 and S2 demonstrated that, in a semi-static model of biofilm formation, S1 forms significantly thicker biofilms with more biomass than S2 (Fig 5). This coincides with lower expression levels of *bfmR* and *bfmS* genes (S3 Table) in S2. BfmS is part of the two-component regulatory systems named BfmR/S (PA4101/PA4102) that regulates biofilm maturation in *P*. *aeruginosa*. BfmR/S has been described as essential for biofilm maturation in *P*. *aeruginosa* by limiting bacteriophage-mediated lysis and thus, eDNA release[65;66]. Other research indicates that BfmS controls the Rhl quorum-sensing system by repressing BfmR, which directly reduces production of quorum-sensing molecules (C4-HSL) by suppressing expression of *rhlR*. Deletion of *bfmS* causes substantially reduced virulence in lettuce leaf, reduced cytotoxicity, enhanced invasion, and reduced bacterial survival during acute mouse lung infection[67]. However, BfmS is able to switch its function from repressor of BfmR to activator through point mutations found in CF-related strains such as the Danish epidemic clone (DK2)[67]. This suggests that under certain conditions reduced expression BfmS and BfmR may indeed coincide with the observed reduced biofilm maturation in S2 relative to S1.

At this moment, however, we can only speculate why loss of biofilm maturation is beneficial in the CF lung. Also, in vitro experiments with surface-attached biofilms may not reflect in the in vivo situation in CF lungs. It has been reported before that other “cooperative traits” like secretion of pyoverdin, elastase, protease and some quorum-sensing molecules are also down-regulated in chronic CF isolates, indicating loss of social behavior of isolates that profit from products produced by cooperative strains[68]. In their study, Jiricny and co-workers did not observe that loss of social behavior also resulted in lower-level biofilm formation, but possibly reduced biofilm formation is advantageous at certain stages of chronic infection of the CF lung by saving energy or increasing transmissibility.

**Fig 6:**
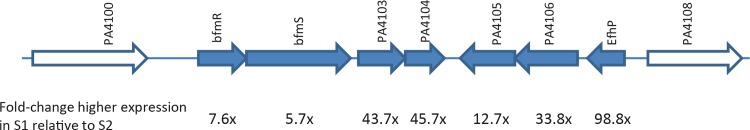
Overview of the gene cluster encompassing genes PA4101-4107. Genes PA4101-4107 are depicted in blue with differences in transcription level between S1 and S2 and adjacent genes in white. Fold changes in expression level in S1 relative to S2 are indicated underneath. PA4103 is a hypothetical gene encoding a putative ferric reductase transmembrane component superfamily, PA4104 is a hypothetical gene encoding a polypeptide with similarities to the DoxX superfamily, PA4105 and PA4106 are hypothetical genes encoding DUF 2063 and DUF 692 superfamily protein, respectively. *EfhP* is the gene originally named PA4107. Arrows indicate direction of transcription.

Directly adjacent to *bfmRS* is a cluster of 5 genes (*PA4103*-*PA4107*) that is, like *bfmRS*, also significantly lower expressed in S2 (Fig 6). It is known that BfmR directly binds to its own and to the promoters of PA4107, PA4103, and *rhlR* and that expression of the operons PA4103-Pa4104 and PA4105-PA4107 is upregulated in CF lungs due to BfmRS activation[67]. The function of these genes is largely unknown with the exception of *PA4107*, which was recently renamed *efhP* and found to be important for Ca^2+^ homeostasis and virulence[69]. Levels of Ca^2+^ are increased in CF lungs[70] and in the presence of high Ca^2+^ strains lacking functional EfhP were unable to produce pyocyanin, developed less biofilm, and had decreased resistance to oxidative stress (H2O2).

Besides differences in colony morphology and biofilm formation as described above, other phenotypic characteristics like swimming, swarming, twitching, production of quorum-sensing molecules and growth rate were comparable between S1 and S2 (Table 3). Protease production was slightly higher in S2. Both strains displayed a slow growing phenotype with lack of motility and lower protease production, compared to PA01. This is something that was also reported for other CF adapted strains[68;71;72]. Both S1 and S2 did not exhibit a mucoid phenotype, which is not unexpected. In general, chronically infected CF patients harbor mucoid *P*. *aeruginosa*, however, mucoidity can be lost by secondary site mutations and mucoid and non-mucoid isolates are often found to co-exist in chronic lung infections[73]. Known virulence-associated phenotypes that are important for *P*. *aeruginosa* colonization and infection, like motility and quorum-sensing are not displayed in both the early and late ST406 isolate. Reduced virulence in CF adapted strains is also described in other studies[74;75].

**Table 3:** Phenotypic traits of S1 and S2 compared to PA01.

phenotypic assay	S1	S2	PA01
swim	+	+	++
swarm	-	-	++
twitch	-	-	++
protease	+	++	+++
quorum sensing	-	-	+
growth rate (doubling time in min)	50	46	29

**Fig 7:**
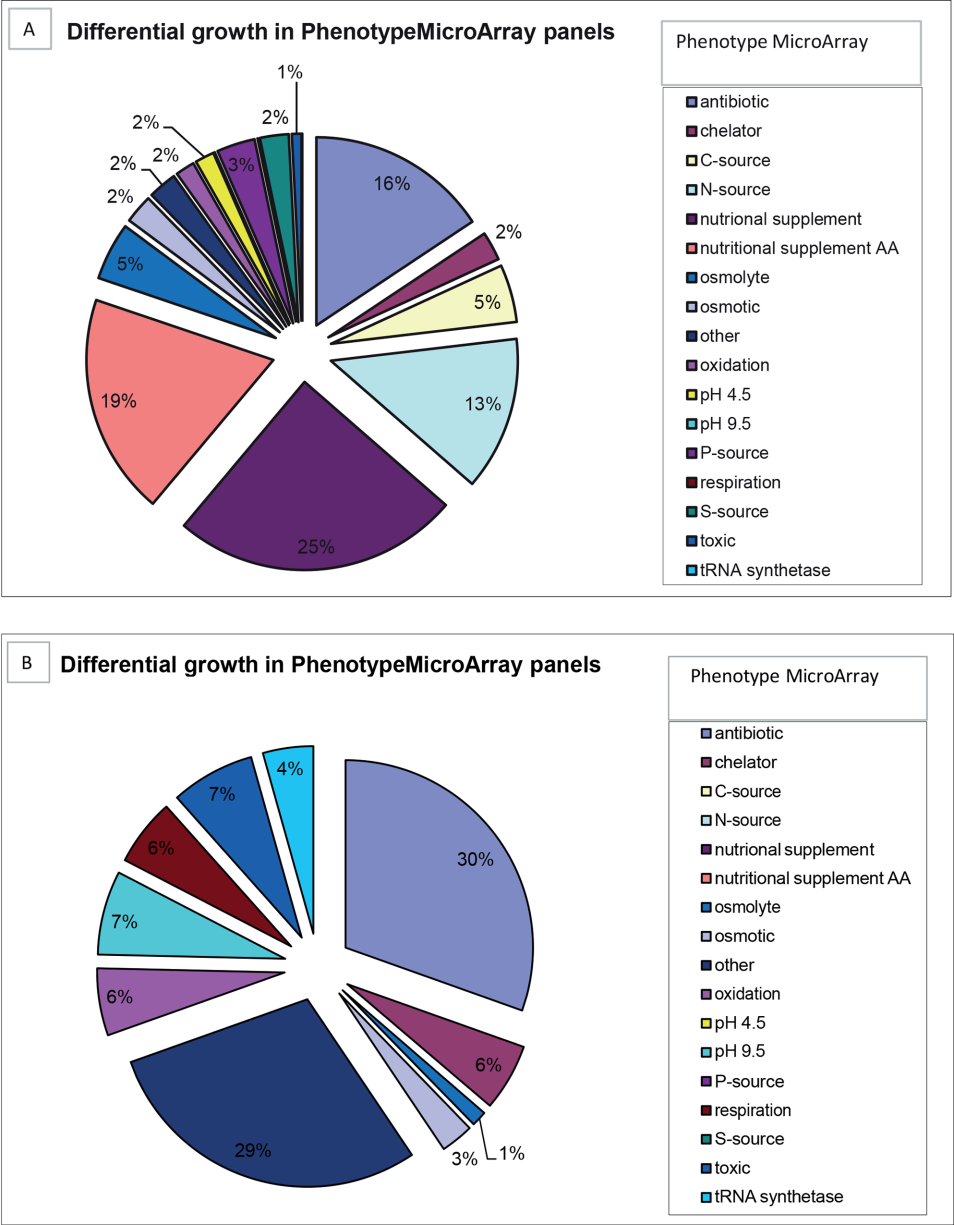
Phenotype MicroArray analysis of the early (S1) and late (S2) ST406 isolate. Indicated are the Phenotype MicroArray panels for which at least a two-fold difference in metabolism (= respiration) between S1 and S2 was detected. A, indicate the percentage of Phenotype MicroArray panels for which the growth rate of S2 was at least two-fold lower than that of S1, while B indicates the percentage of Phenotype MicroArray panels for which the growth rate of S2 was at least two-fold higher than that of S1. Phenotype MicroArray panel categories are color-coded and indicated on the right (C = carbon, S = Sulphur, N = Nitrogen, P = Phosphor).

To further quantitatively measure phenotypic traits of S1 and S2, growth rates of S1 and S2 were measured on different substrates using Phenotype MicroArrays. Relative to S1, S2 demonstrated enhanced metabolism in the presence of toxic substances like chloride and bromide detergents, and diverse oxidizing agents, while the S2 isolate displayed decreased growth capabilities on various nutritional supplements like several amino acids and different Carbon (1,2-Propanediol, Adenosine, D-Arabitol, D-Glucosaminic Acid, D-Glucuronic Acid, L-Alaninamide), Phosphor (Dithiophosphate, D-Mannose-6-Phosphate, O-Phospho-D-Serine, O-Phospho-L-Serine) and Nitrogen sources (Fig 7 and S5 Table). This indicated that the transition from early to late isolate was accompanied with major metabolic changes. Higher expression of genes involved in metabolism in S1 from transcriptome data matches the phenotypic microarray data. Metabolic changes have also been described before in chronic CF strains[14].

### Differences in gene content between the early (S1) and late (S2) isolate

**Fig 8:**
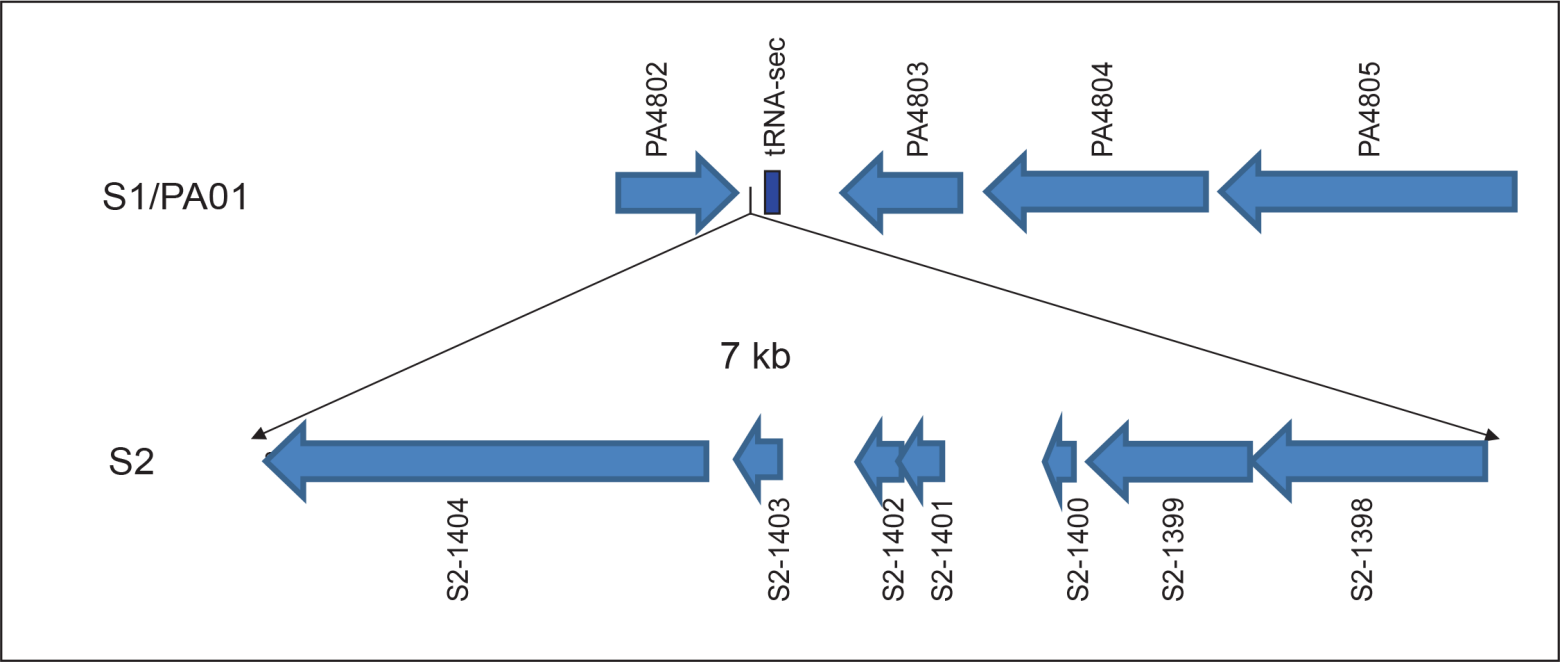
Overview of the seven gene cluster present in the S2 ST406 isolate, but not in the S1 isolate. The 7 kb insert next to tRNAsec and upstream of gene PA4802 detected only in S2 includes a phage integrase gene (S2-1398) and a gene encoding a putative replication initiation factor (S2-1399) and five genes encoding hypothetical proteins.

Annotation of the S1 and S2 genomes revealed 5766 protein-encoding genes in S1 and 5772 protein-encoding genes in S2. This cluster of seven genes in S2 was absent in S1. This means that either this cluster was acquired by S2 during chronic colonization or that this insert was already present in the *P*. *aeruginosa* (sub-) population at the early time point when strain S1 was sampled. These seven genes encode five hypothetical proteins, a putative replication initiation factor and a phage integrase, suggesting incorporation of phage-associated genes in S2. This seven-gene cluster is located next to a tRNA-SeC (p)-TCA gene and does not seem to disrupt a coding region or a predicted operon. These features may suggest that this seven-gene region represents a mobile genetic element, although we have no formal proof for this. On both sides of the 7 genes there is an identical stretch of 36 bp. This partly overlaps with the tRNA gene on the 3’ side (Fig 8). PCR performed on three targets of this 7 kb DNA islet respectively including part of predicted genes S2-1398/1399 (893 bp), S2-1401/1402 (426 bp) and S2-1404 (399 bp), in 29 non-ST406 clinical isolates (6 ICU, 6 community acquired, 6 CF, 6 other clinical isolates from a previous study[26] and 3 isolates of the Liverpool epidemic strain, PA01 and the Midlands epidemic strain) did not result in a positive PCR product, indicating that this insertion is specific for the ST406 S2 and, therefore, has probably no general role in adaptation of *P*. *aeruginosa* to the lung of CF patients.

## Conclusion

This study characterized genome-wide and phenotypic changes that have occurred in the Dutch high-prevalent clone ST406 after three years of chronic carriage. The data demonstrate that within-host evolution of the Dutch ST406 clone within a CF patient during three years was not driven by major gene acquisition or gene loss, but mostly by point mutations that coincided with differences in transcriptome levels and phenotype, most notably with differences in biofilm formation. These adaptive evolutionary events in ST406 partly overlap with adaptations described for other chronic CF isolates. In general, clone ST406 seems to have a phenotypic profile of low virulence. Drawing firm conclusions on diversification over time are limited by the fact that in this study only one colony per sputum cultured was used and diversity within the *P*. *aeruginosa* population the CF lung at the same time point have been demonstrated[7;13;51–53]. Since the early isolate was recovered from a CF patient just one month after first time colonization with *P*. *aeruginosa*, diversification at that time was probably low, but the single late isolate might not capture the full diversity of *P*. *aeruginosa* isolates present at that time point capture the full diversity of *P*. *aeruginosa* isolates present at that time point [53]. The low virulence profile of the early isolate suggests that at the time of isolation it had already undergone a certain level of adaptation to the CF lung. Common adaptive changes that are repeatedly found in chronically infecting isolates may provide novel leads for targeted therapeutic interventions to combat chronic colonization with *P*. *aeruginosa* in CF patients.

## Supporting Information

S1 Table Primers and strains used for S2 specific gene amplification. (DOCX)

S2 Table Published strains used in SNP-based phylogenetic tree. (DOCX)

S3 Table Differential expressed genes between early and late isolate by Affymetrix (p<0.05 and >2fold change. (DOCX)

S4 Table Antibiotic susceptibility of S1 and S2. (DOCX)

S5 Table Phenotype MicroArray panels for which at least a two-fold difference in metabolism (= respiration) between S1 and S2 was detected. (XLSX)
