# Supplementary material for: Within-Host Evolution of the Dutch High-Prevalent Pseudomonas aeruginosa Clone ST406 during Chronic Colonization of a Patient with Cystic Fibrosis
Source: PLoS One. 2016 Jun 23;11(6):e0158106. doi: 10.1371/journal.pone.0158106 (PMC4918941; doi:10.1371/journal.pone.0158106)
Supplement: S1 Table — (DOCX) [file pone.0158106.s001.docx]

**S1 Table.** primers and strains used for S2 specific gene amplification

| **Strain code** | **Primer sequence** |
| --- | --- |
| S2 1401/1402 lower | GCA GGG AGC ACG ACA CCG ACG AC |
| S2 1401/1402 upper | CGT CCC ACA AGG TCG GCA AAC ACA |
| S2 1404 lower | TAC ACC GCC AGC CTT CCT CGT T |
| S2 1404 upper | TGA CAT CGA AAG CGC CAT TAC AGA |
| S2 1398/1399 lower | AGC TTC AGC GGG CGG ACC TTT TC |
| S2 1398/1399 upper | TGA TCG CCA CGC AGA CCT ACG |
|  |  |
| LES431 | kindly provided by Craig Winstanley |
| LES400 | kindly provided by Craig Winstanley |
| LES58B | kindly provided by Craig Winstanley |
| Midlands 1 | purchased from HPA |
| PA01 | kindly provided by Søren Molin |
| U-1_10 | ICU strain from previous study* |
| U-1_16 | ICU strain from previous study* |
| U-1_17 | ICU strain from previous study* |
| U-1_23 | ICU strain from previous study* |
| U-1_26 | ICU strain from previous study* |
| U-1_29 | ICU strain from previous study* |
| ZH829 | community acquired strain from previous study* |
| ZH836 | community acquired strain from previous study* |
| ZH843 | community acquired strain from previous study* |
| ZH846 | other clinical isolate from previous study* |
| ZH852 | other clinical isolate from previous study* |
| ZH853 | other clinical isolate from previous study* |
| ZH857 | other clinical isolate from previous study* |
| ZH870 | other clinical isolate from previous study* |
| ZH879 | other clinical isolate from previous study* |
| ZH944 | community acquired strain from previous study* |
| ZH956 | community acquired strain from previous study* |
| ZH979 | community acquired strain from previous study* |
| PA208 | CF strain from previous study* |
| PA212 | CF strain from previous study* |
| PA214 | CF strain from previous study* |
| PA271 | CF strain from previous study* |
| VW186 | CF strain from previous study* |
| VW395 | CF strain from previous study* |

* REF 25: van Mansfeld *et al*, PLoSOne 2010.
