# Supplementary material for: Within-Host Evolution of the Dutch High-Prevalent Pseudomonas aeruginosa Clone ST406 during Chronic Colonization of a Patient with Cystic Fibrosis
Source: PLoS One. 2016 Jun 23;11(6):e0158106. doi: 10.1371/journal.pone.0158106 (PMC4918941; doi:10.1371/journal.pone.0158106)
Supplement: S2 Table — (DOCX) [file pone.0158106.s002.docx]

**S2 Table.** Published strains used in SNP-based phylogenetic tree

| **strain** | **origin^a^** | **Country^a^** | **year^a^** |
| --- | --- | --- | --- |
| 2192 | chronic CF | Boston USA |  |
| 39016 | cornea, ulcerative keratitis | GB | 2003 |
| 138244 | sputum, pneumonia | Portugal | 2011 |
| 152504 | sputum | Portugal | 2011 |
| 18A | CF non clonal |  |  |
| ATCC 14886 | soil |  |  |
| ATCC 700888 | biofilm, industrial water system |  |  |
| B13633 | diarrea community acquired, child | China |  |
| C3719 | CF Manchester epidemic strain | Gr Britain |  |
| CI27 | chronic CF |  |  |
| CIG1 | chronic CF |  |  |
| DK2 | CF chronic | Denmark | 2007 |
| E2 | tomato plant | Florida, USA |  |
| LCT PA102 | ATCC 27853 |  |  |
| LES431 | parent of CF patient | Great Br |  |
| LESB58 | chronic CF Liverpool epidemic strain | Great Br |  |
| M18 | rhizosphere watermelon |  |  |
| MTB | contaminated soil | India |  |
| NCGM2 S1 | urinary tract ingfection MDR outbr | Japan |  |
| NCMG1179 | resp tract, MDR outbreak med faci | Japan | 2010 |
| PA01 | wound, laboratory strain | Australia | 1955 |
| PA0579 | mutant of PA01 | Australia | 1975 |
| PA07 | clinical, non-respiratory | Argentina |  |
| PA1 |  | military hosp |  |
| PA1R |  | military hosp |  |
| PA21 ST175 | blood, VIM+, outbreak strain | Spain |  |
| PAb1 | frostbite clinical sample | USA |  |
| PACS2 | CF 6 months | Canada |  |
| PADK2CF510 | CF chronic | Denmark |  |
| RP73 | CF chronic (16,9 yrs) |  |  |
| S1 | CF first isolate | Netherlands | 2004 |
| S2 | CF chronic | Netherlands | 2007 |
| S3 | CF chronic | Netherlands | 2007 |
| SCV20265 | CF small colony variant, MDR | Italy | 2006 |
| SJTD 1 | soil | China |  |
| UCBPP PA 14 | human burn patient |  |  |
| XMG | soil | China | 2012 |
| VRFPA01 | blood | India |  |
| MPAO1_P1 | PA01 phenotype 1 |  |  |
| MPAO1_P2 | PA01 phenotype 2 |  |  |

^a^ indicated when available as published on NCBI website
