# Supplementary material for: Within-Host Evolution of the Dutch High-Prevalent Pseudomonas aeruginosa Clone ST406 during Chronic Colonization of a Patient with Cystic Fibrosis
Source: PLoS One. 2016 Jun 23;11(6):e0158106. doi: 10.1371/journal.pone.0158106 (PMC4918941; doi:10.1371/journal.pone.0158106)
Supplement: S3 Table — (DOCX) [file pone.0158106.s003.docx]

**S3 Table**. Differential expressed genes between early and late isolate by Affymetrix (p<0.05 and >2fold change

| **locus-tag** | **COG functionclassification** | **pvalue** | **ratio S1/S2** | **gene** |
| --- | --- | --- | --- | --- |
| PA4489 | Adaptation, Protection | 0.01 | 0.43 | magD |
| PA2536 | Amino acid transport and metabolism | 0.02 | 0.34 |  |
| PA2290 | Carbohydrate transport andmetabolism | 0.03 | 0.48 | gcd |
| PA0070 | Cell wall/membrane/envelope biogenesis | 0.01 | 0.25 | tagQ1 |
| PA0045 | Cell wall/membrane/envelope biogenesis | 0.01 | 0.29 |  |
| PA2684 | Cell wall/membrane/envelope biogenesis | 0.02 | 0.30 |  |
| PA2457 | Cell wall/membrane/envelope biogenesis | 0.02 | 0.46 |  |
| PA0072 | Cell wall/membrane/envelope biogenesis | 0.02 | 0.48 | tagS1 |
| PA2454 | Coenzyme transport and metabolism | 0.03 | 0.44 |  |
| PA0073 | Defense mechanisms | 0.03 | 0.34 | tagT1 |
| PA0046 | Function unknown | 0.01 | 0.26 |  |
| PA0047 | Function unknown | 0.02 | 0.26 |  |
| PA0563 | Function unknown | 0.01 | 0.27 |  |
| PA3021 | Function unknown | 0.01 | 0.35 |  |
| PA3729 | Function unknown | 0.01 | 0.35 |  |
| PA0093 | Function unknown | 0.03 | 0.39 |  |
| PA4490 | Function unknown | 0.02 | 0.43 | magC |
| PA0094 | Function unknown | 0.04 | 0.45 |  |
| PA4492 | Function unknown | 0.04 | 0.46 | magA |
| PA4491 | Function unknown | 0.03 | 0.48 | magB |
| PA2455 | General function prediction only | 0.01 | 0.40 |  |
| PA0074 | General function prediction only, Signal transduction mechanisms, Transcription, Replication, recombination and repair | 0.01 | 0.33 | ppkA |
| PA0087 | Hcp secretion island I (HSI-I) T6SS | 0.00 | 0.21 | tssE1 |
| PA0078 | Hcp secretion island I (HSI-I) T6SS | 0.00 | 0.22 | tssL1 |
| PA0080 | Hcp secretion island I (HSI-I) T6SS | 0.00 | 0.22 | tssJ1 |
| PA0082 | Hcp secretion island I (HSI-I) T6SS | 0.00 | 0.23 | ttsA1 |
| PA0086 | Hcp secretion island I (HSI-I) T6SS | 0.00 | 0.23 | tagJ1 |
| PA0085 | Hcp secretion island I (HSI-I) T6SS | 0.01 | 0.24 | hcp1 |
| PA0076 | Hcp secretion island I (HSI-I) T6SS | 0.01 | 0.25 | tagF1 |
| PA0077 | Hcp secretion island I (HSI-I) T6SS | 0.00 | 0.25 | icmF1 |
| PA0084 | Hcp secretion island I (HSI-I) T6SS | 0.02 | 0.26 | ttsC1 |
| PA0088 | Hcp secretion island I (HSI-I) T6SS | 0.00 | 0.27 | tssF1 |
| PA0089 | Hcp secretion island I (HSI-I) T6SS | 0.01 | 0.27 | tssG1 |
| PA0079 | Hcp secretion island I (HSI-I) T6SS | 0.01 | 0.29 | tssK1 |
| PA0083 | Hcp secretion island I (HSI-I) T6SS | 0.02 | 0.30 | ttsB1 |
| PA1844 | Hcp secretion island I (HSI-I) T6SS | 0.02 | 0.30 | tse1 |
| PA2685 | Hcp secretion island I (HSI-I) T6SS | 0.01 | 0.32 | vgrG4 |
| PA3484 | Hcp secretion island I (HSI-I) T6SS | 0.04 | 0.35 | tse3 |
| PA0071 | Hcp secretion island I (HSI-I) T6SS | 0.04 | 0.45 | tagR1 |
| PA0126 | Hypothetical, unclassified, unknown | 0.01 | 0.24 |  |
| PA3716 | Hypothetical, unclassified, unknown | 0.01 | 0.34 |  |
| PA2781 | Hypothetical, unclassified, unknown | 0.02 | 0.37 |  |
| PA3661 | Hypothetical, unclassified, unknown | 0.02 | 0.40 |  |
| PA3850 | Hypothetical, unclassified, unknown | 0.02 | 0.42 |  |
| PA5441 | Hypothetical, unclassified, unknown | 0.04 | 0.42 |  |
| PA2456 | Hypothetical, unclassified, unknown | 0.02 | 0.44 |  |
| PA2792 | Hypothetical, unclassified, unknown | 0.01 | 0.45 |  |
| PA3483 | Hypothetical, unclassified, unknown | 0.01 | 0.45 |  |
| PA3485 | Hypothetical, unclassified, unknown | 0.02 | 0.47 |  |
| PA1639 | Hypothetical, unclassified, unknown | 0.02 | 0.48 |  |
| PA2540 | Lipid transport and metabolism | 0.01 | 0.30 |  |
| PA2537 | Lipid transport and metabolism | 0.02 | 0.31 |  |
| PA2541 | Lipid transport and metabolism | 0.03 | 0.46 |  |
| PA3727 | Lipid transport and metabolism | 0.01 | 0.47 |  |
| PA4317 | Membrane proteins | 0.01 | 0.27 |  |
| PA5113 | Membrane proteins | 0.01 | 0.33 |  |
| PA2538 | Membrane proteins | 0.02 | 0.35 |  |
| PA2774 | Membrane proteins | 0.05 | 0.39 |  |
| PA5114 | Membrane proteins | 0.05 | 0.44 |  |
| PA3730 | Membrane proteins | 0.02 | 0.45 |  |
| PA2775 | Membrane proteins | 0.02 | 0.47 |  |
| PA4318 | Membrane proteins | 0.03 | 0.47 |  |
| PA0277 | Posttranslational modification, protein turnover, chaperones | 0.01 | 0.20 |  |
| PA0090 | Posttranslational modification, protein turnover, chaperones | 0.01 | 0.24 | clpV1 |
| PA1791 | Posttranslational modification, protein turnover, chaperones | 0.02 | 0.34 |  |
| PA1069 | Posttranslational modification, protein turnover, chaperones | 0.02 | 0.43 |  |
| PA0091 | Protein secretion/export apparatus | 0.01 | 0.32 | vgrG1 |
| PA1202 | Secondary metabolites biosynthesis, transport and catabolism | 0.01 | 0.14 |  |
| PA2539 | Signaltransduction mechanisms | 0.02 | 0.27 |  |
| PA0075 | Signaltransduction mechanisms | 0.02 | 0.28 | pppA |
| PA0081 | Signaltransduction mechanisms | 0.03 | 0.36 | fha1 |
| PA2432 | Transcription | 0.00 | 0.19 | bexR |
| PA2780 | Transcription | 0.04 | 0.36 |  |
| PA3267 | Transcription | 0.03 | 0.46 |  |
| PA0495 | Amino acid transport and metabolism | 0.04 | 2.68 |  |
| PA0609 | Amino acid transport and metabolism, Coenzyme transport and metabolism | 0.02 | 3.06 | trpE |
| PA2321 | Carbohydrate transport and metabolism | 0.04 | 2.10 |  |
| PA2493 | Cell wall/membrane/envelope biogenesis | 0.00 | 3.52 | mexE |
| PA2494 | Defense mechanisms | 0.03 | 2.53 | mexF |
| PA4770 | Energy production and conversion | 0.05 | 2.71 | lldP |
| PA4105 | Function unknown | 0.00 | 12.71 |  |
| PA4106 | Function unknown | 0.00 | 33.81 |  |
| PA4104 | Function unknown | 0.00 | 45.67 |  |
| PA4107 | Function unknown | 0.00 | 98.76 |  |
| PA0492 | General function predictiononly | 0.04 | 3.46 |  |
| PA3843 | Hypothetical, unclassified, unknown | 0.02 | 2.56 |  |
| PA3057 | Hypothetical, unclassified, unknown | 0.01 | 2.73 |  |
| PA4881 | Hypothetical, unclassified, unknown | 0.02 | 2.91 |  |
| PA3229 | Hypothetical, unclassified, unknown | 0.00 | 3.73 |  |
| PA4103 | Hypothetical, unclassified, unknown | 0.00 | 43.68 |  |
| PA4358 | Inorganic ion transport and metabolism | 0.02 | 2.18 |  |
| PA4359 | Inorganic ion transport and metabolism | 0.02 | 2.50 |  |
| PA3790 | Inorganic ion transport and metabolism | 0.00 | 3.55 | oprC |
| PA1707 | Intracellular trafficking, secretion, and vesicular transport | 0.03 | 2.59 | pcrH |
| PA0494 | Lipid transport and metabolism | 0.05 | 3.24 |  |
| PA0493 | Lipid transport and metabolism | 0.03 | 4.29 |  |
| PA1705 | Protein secretion/export apparatus, T3SS | 0.04 | 2.14 | pcrG |
| PA1709 | Protein secretion/export apparatus, T3SS | 0.04 | 2.16 | popD |
| PA1706 | Protein secretion/export apparatus, T3SS | 0.02 | 2.19 | pcrV |
| PA1696 | Protein secretion/export apparatus, T3SS | 0.02 | 2.31 |  |
| PA1701 | Protein secretion/export apparatus, T3SS | 0.01 | 2.64 | pcr3 |
| PA1708 | Protein secretion/export apparatus, T3SS | 0.01 | 3.12 | popB |
| PA3842 | Protein secretion/export apparatus, T3SS | 0.01 | 4.30 | spcS |
| PA3841 | Signaltransduction mechanisms | 0.00 | 2.66 | exoS |
| PA4102 | Signaltransduction mechanisms | 0.00 | 5.69 | bfmS |
| PA4101 | Signaltransduction mechanisms, Transcription | 0.01 | 7.61 | bfmR |
| PA3056 | Transcription | 0.04 | 2.01 |  |
| PA3055 | Transcription | 0.02 | 2.25 |  |
