# Supplementary material for: Within-Host Evolution of the Dutch High-Prevalent Pseudomonas aeruginosa Clone ST406 during Chronic Colonization of a Patient with Cystic Fibrosis
Source: PLoS One. 2016 Jun 23;11(6):e0158106. doi: 10.1371/journal.pone.0158106 (PMC4918941; doi:10.1371/journal.pone.0158106)
Supplement: S4 Table — (DOCX) [file pone.0158106.s004.docx]

**S4 Table.** Antibiotic susceptibility of S1 and S2

| Antibiotics | Diameter agar diffusion in mm^a^ | |
| --- | --- | --- |
|  | S1 | S2 |
| ciprofloxacin | 19 (R) | 19 (R) |
| levofloxacin | 11 (R) | 14 (R) |
| piperacillin | 26 (S) | 28 (S) |
| ceftazidime | 25 (S) | 23 (S) |
| tobramycin | 18 (S) | 14 (R) |
| amikacin | 17 (I) | 15 (I) |
| meropenem | 27 (S) | 35 (S) |
| piperacillin/tazobactam | 32 (S) | 35 (S) |
| colistin* | 24 | 23 |
| trimethoprim* | 0 | 0 |
| trimethoprim/sulfamethoxazole* | 0 | 20 |
| chloramphenicol* | 0 | 17 |

^a^ Resistance breakpoints for R, I, and S were defined according to EUCAST guidelines

* no resistance breakpoint available in EUCAST for disk distribution
